# Arthroscopic knotless anterior labral stabilization using labral tape and wide awake anaesthesia-short term results

**DOI:** 10.1186/s12891-018-2164-x

**Published:** 2018-07-18

**Authors:** John Edwin, Daniel Morris, Shahbaz Ahmed, Paul Townsley, Paul Manning, Benjamin Gooding

**Affiliations:** 1 Circle Nottingham NHS Treatment Centre, Lister Road, Nottingham, NG7 2FT UK; 2 Basildon and Thurrock University Hospitals NHS Trust, Nethermayne, Basildon, SS16 5NL UK; 3 Sidcup, 73, Faraday Avenue, Nottingham, DA14 4JB UK

**Keywords:** Anterior instability, Knotless, Suture tape, Awake anaesthesia

## Abstract

**Background:**

The shoulder is the least constrained of all joints of the body and is more susceptible to injury including dislocation. The rate of recurrent instability following primary stabilization procedure at 10 years of follow-up ranged from 3.4 to 20%. There is a lack of evidence in the literature regarding use of labral tape and anchors for anterior stabilization despite the growing market for this product. We describe the outcomes of 67 patients who underwent knotless arthroscopic anterior stabilisation under awake anaesthesia using 1.5 mm LabralTape with 2.9 mm Pushlock anchors for primary anterior instability by a single surgeon.

**Methods:**

This was a retrospective analysis of prospectively collected outcome data for adult patients undergoing anterior stabilisation for primary traumatic anterior shoulder instability between 2013 and 2016 at two centres. Patients with > 25% glenoid bone loss, engaging Hill Sach’s, and multidirectional instability were excluded. All cases underwent surgery using awake anaesthetic technique. The surgical technique and post-operative physiotherapy was standardized. Outcomes were measured at 6 months and 12 months.

**Results:**

Of the 74 patients in our study, 7 were lost to follow up. Outcomes were measured using the Oxford Instability Shoulder Score (OISS) and clinical assessment including the range of motion. The OISS showed statistically significant improvement from a mean score and standard deviation (SD) of 24.72 ± 2.8 pre-surgery to 43.09 ± 3.5 after the procedure at 12 months with good to excellent outcomes in 66 cases (98.5%). The mean abduction was 134.2 ± 6.32 and external rotation was 72.55 ± 5.42 at 60–90 position at 12 months. We report no failures due to knot slippage or anchor pull-out.

**Conclusion:**

Our case series using the above technique has distinct advantages of combining a small non-absorbable implant with flat, braided, and high-strength polyethylene tape. This technique demonstrates superior medium term results to conventional suture knot techniques for labral stabilization thereby validating its use.

## Background

Shoulder dislocation is commonly associated with injuries due to sports and can potentially be disabling. It is the least constrained joint of the human body [1]. Stability of the shoulder joint is a summation of interplay between normal static and dynamic stabilizers working cohesively. The ball and socket configuration of the shoulder joint with the increased depth achieved from the labral attachment provides an increased range of mobility at the cost of stability. Mechanical injuries can involve the soft tissues and bone. If left untreated, this often leads to recurrence and resultant instability. The biomechanics of shoulder instability has been well described [2] and is often multifactorial. The Bayley or Stanmore triangle [3] describes the causes of instability into three subgroups and attempts to provide options of management in these cases. Upto 97% of shoulder dislocations are anterior and most commonly due to trauma with incidence of 8.2 to 23.9 per 100,000 per year [4–6]. Surgical treatment for anterior shoulder instability has progressed rapidly over the past 2 decades. Further advances in diagnostic imaging have led to a better understanding of the pathology involved. Different treatment pathways have been postulated for acute and recurrent anterior instability depending on the incidence, age, sex, size and location of the soft tissue/bony lesion (Bankart/ Hill- Sachs lesion) [7]. The mainstay of surgical management for recurrent anterior shoulder instability with a Bankart lesion is arthroscopic Bankart repair with proven excellent results [8]. Despite the advances in arthroscopic techniques, significant complications related to the implant and suture material includes implant failure, knot slippage and chondral damage [9]. Our study aims to review our results of arthroscopic anterior stabilization with knotless 1. 5 mm suture tape (LabralTape, Arthrex, Naples, Fl) coupled with 2.9 mm suture anchors (PEEK PushLock, Arthrex, Naples, Fl) using awake anaesthetic technique. We hypothesized that the use of knotless anchor and suture tape in combination provides good results comparable to other techniques documented in the literature.

## Methods

We reviewed the results of 74 adult patients who underwent primary arthroscopic anterior stabilization by a single surgeon for primary traumatic anterior instability with recurrent symptoms from 2013 to 2016 at two institutions with a shared medical record. Prior clinical governance approval was obtained for this service evaluation. We retrospectively analysed prospectively collected preoperative and post-operative outcome at 6 and 12 months. Patients with > 25% glenoid bone loss, an engaging Hill Sachs lesion, posterior dislocation, and multidirectional instability were excluded. Initial clinical evaluation included assessment for pain, range of movement, the load and shift test and positive apprehension tests. In addition, all patients had plain radiographs of the shoulder and additional assessment regarding suitability of the procedure was done by MRI or at the time of arthroscopy. We assessed outcomes at follow-up measuring the post-operative range of motion, function, return to pre injury activity and using the Oxford Instability Shoulder Score (OISS). The OISS is an objective 12 point questionnaire measuring the patient’s activity on points based system with worse outcome measuring 0 and the best outcome measuring 48 points. The results were explained as poor outcomes 0–19, fair 20–29, good 30–39 and excellent 40–48. Statistical analysis of pre and post-operative OISS was performed using SPSS (SPSS Inc. SPSS for Windows, Version 17.0. Chicago: SPSS Inc.) and a paired, two tailed Student’s T test. Statistical significance was set at 5%. Recurrent dislocation/ sensation of instability, directly attributable pain with positive apprehension test and failure to return to functional pre injury mobility were considered as failure of the procedure.

## Procedure

All procedures were performed by a single surgeon using a standardized protocol for admission, intraoperative technique, post-operative management and physiotherapy regime during this period. Following preoperative consultant review, all cases underwent surgery using the awake anaesthetic technique. This involved ultrasound-guided interscalene brachial plexus blockade with 10–15 ml of 1% Ropivacaine and selective blockade of the supraclavicular branches of the superficial cervical plexus. Intra-operatively the patients either stayed awake or received conscious sedation using midazolam and target controlled propofol infusion [10]. Additional analgesia was provided with 0.5% Marcaine with 1:200,000 adrenaline at the posterior portal site just prior to the start of the procedure. All patients were consented for the possibility (5%) of conversion to either intravenous analgesia or general anaesthesia. Surgery was performed as a day case procedure and patients were discharged with multimodal analgesia comprising paracetamol, ibuprofen and oral morphine to take when the nerve block wore off. The procedure was performed in the standard 70 degree reclining beach chair position. A posterior viewing portal was created approximately 2 cms inferior and medial to the angle of the acromion as the anatomic landmark. The anterosuperior portal was established through the rotator interval as the working portal. A standard sized flexible cannula was inserted through this portal to facilitate ease of passing instruments and implants. The glenohumeral joint was visualised in a systematic manner and the labral detachment noted. The glenoid defect is noted, if any. The torn labrum was detached from the anterior glenoid neck using 30 degree arthroscopic soft tissue elevator. Care was taken to avoid tearing through the labral tissue. Sufficient mobilization was indicated when the subscapularis was visible through the elevated labrum. Another test to confirm the same was to shut off the fluid inflow into the joint temporarily and visualise if the labrum settled at the edge of the glenoid instead of the neck. The glenoid margins were freshened using the rasp to create a fresh bleeding surface. Using a curved suture passer, the first suture using the tape is shuttled through the capsulolabral tissue at the 5.30 position. Placement of this suture is most important as this helps to tighten and elevate the inferior glenohumeral ligament (IGHL) complex. This was ideally done by penetrating the tip of the suture passer approximately 1 cm inferiorly through the IGHL prior to passing through the labrum. A drill hole using the pre-packed drill bit and drill guide at the 5:30 position on the glenoid is made. This drill hole is ideally placed *en-face* on the glenoid to create the bumper effect and also to augment the concavity and negative pressure within the joint after the repair. We used suture tape in all our cases. This is ideal as it is a smooth, low profile, made from polyethylene, wider than a traditional suture thus provides a larger area of compression and less chance of suture cut-out. The tape was loaded onto the anchor and passed through the same anterosuperior portal to engage into the drill hole. The ends of the suture tape are gently tensioned till the labrum was held firmly onto the glenoid near the eyelet of the anchor before hitching the ends onto the cleats of the anchor inserter. Care was taken to avoid overtightening or strangulating the labrum at this point. The inserter was gently tapped through till the laser mark was at the level of the drill hole indicating sufficient depth of insertion. It is important to avoid penetration of the anchor through the inferior glenoid. The suture tape is locked within the anchor before removing the inserter. The sutures were cut flush to the labrum. Additional anchors were placed as required using the same technique. Tightening of the inferior sling is confirmed by a negative drive-through sign.

All our patients were discharged on the same day with instructions on mobilization and pain management. Follow up was performed by extended scope physiotherapists within a multidisciplinary clinic. This clinic was indirectly supervised by the consultant team. This flexibility allowed follow- up clinic visits to be minimised without compromising on patient care. Post-operative protocol includes 4 weeks of sling use allowing external rotation to neutral followed by active range of motion and passive assisted exercises from 4 to 8 weeks. Impact sports are restricted before 14 weeks. Post-operative scores were done at 6 months and 12 months after surgery.

## Results

**Fig. 1:**
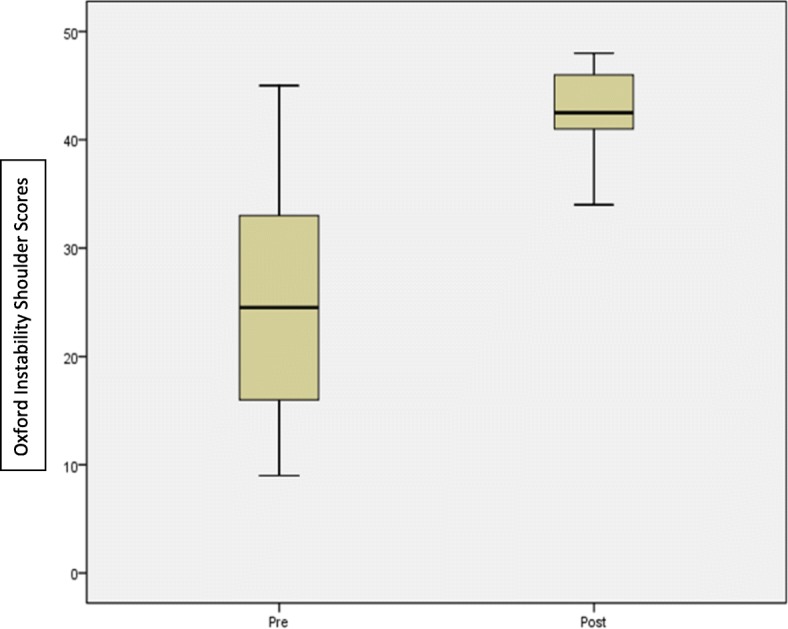
Boxplot diagram demonstrating Pre- and Post-operative Oxford Instability Shoulder Scores. At 12 months post-operatively, the median OISS was 43 suggesting significant improvement of outcomes

Of the 74 patients in our study, we included 67 patients who underwent arthroscopic anterior shoulder stabilization for recurrent anterior shoulder instability. 7 patients were lost to follow up at 12 months. The bio demographics of our study had 60 males and 7 females with mean age 33 years (range 19–57 years). Our patients were included from differing backgrounds with varying levels of demand due to their professional and recreational activities. None of our patients were professional athletes. 29 repairs involved the left shoulder and 38 on the right shoulder. The arthroscopic stabilization involved 2 anchors in 50 cases. 3 anchors were used in 14 cases and a single anchor was used in 3. We measured pre and post-operative outcomes for 67 patients at 6 months and 12 months specifically assessing for stability, range of motion and Oxford Instability Shoulder Score [11]. We noted significant improvement in pain and range of movements in all our patients at the end of 12 months with mean abduction of 134.2 ± 6.32 and external rotation of 72.55 ± 5.42 with the arm in 60–90 position. The OISS showed statistically significant improvement from a mean score and standard deviation (SD) of 24.72 ± 2.8 pre-surgery to 43.09 ± 3.5 after the procedure at 12 months with good to excellent outcomes in 66 cases (98.5%) (Fig. 1). The Pearson correlation was .28. Paired, two tailed Student’s T test demonstrated a statistically significant *p* value of < 0.001. We had no immediate post-surgical complications. Two of our patients sustained mechanical falls during the rehabilitation period and presented with recurrent instability. For one patient further imaging confirmed no internal injury and symptoms resolved with physiotherapy leading to an excellent final outcome on OISS at 12 months. One case continued to experience instability requiring an open bone block stabilisation procedure. A third patient reported some recurrent instability symptoms during rehab although OISS at 12 months defined a good outcome. The true success rate is therefore 95.5%.

## Discussion

Recurrent dislocation of the shoulder is a significant problem especially in the young patient. Sakellarides and Rowe [12] have described higher rates of recurrences in patients who sustain the first dislocation at a younger age group. Symptomatic anterior instability was historically treated with open repair of the labrum prior to the development of arthroscopy. Many studies have demonstrated good results with the open technique with recurrence rate between 0 and 11% and had remained the gold standard till about two decades ago [13, 14]. The main complications related to the open Bankart repair were shoulder stiffness, especially in external rotation and subscapularis dysfunction. Initial arthroscopic techniques used staples to repair the capsulolabral complex which resulted in high failure rate with recurrence in 16–33% [15]. Other techniques such as bioabsorbable tacks and transglenoid suturing had led to further failures [16–19]. Freedman et al. (2004) concluded in their meta-analysis that the results of open Bankart repair was superior compared to the arthroscopy group at the time. This study did not however consider the results of newer arthroscopic techniques of suture anchor fixation and capsular plication [16]. Over the past two decades, a better understanding of soft tissue biology and newer implant materials have seen the progression of bone anchors from metal to PEEK (Poly Ethyl Ethyl Ketone) and Biocomposite materials. Similarly, the suture material used to perform the repair has also undergone significant improvement to stronger, low profile non absorbable materials. Indeed, a recent systematic review comparing open to arthroscopic Bankart repair suggests that there is no significant difference in the failure rates [19]. The results of our study are comparable to the results of open stabilization without the attendant known complications of an open procedure.

Cadaveric studies have concluded the risk of knotted repairs causing movement related knot migration and potential chondral abrasion when the knot becomes interposed in between the articular glenoid and humeral head [20]. The advantages of performing repair with knotless anchors have proven to be a statistically stronger construct under tensile loads [21]. Our use of knotless anchors coupled with 1.5 mm suture tape provides excellent anchor material related properties combining with knotless low profile repair of the labrum.. Biomechanical testing of pull-out strength of our anchor with suture combination is 200 N and this is much more than needed in instability repair. We positioned our knotless anchors *en-face* on the glenoid to restore the labral bumper and create a congruent convex-concave interface between the humeral head and the glenoid-labrum complex. The majority of our patients required 2 anchors on the anterior glenoid *(n = 50)* as the suture we used provided a better tightening of the inferior labroligamentous sling during the capsular shift, in contrast to our previous practice of 3 anchors to achieve the same intraoperative confidence. Only a small number needed three anchors *(n = 14*).

Our awake anaesthesia pathway utilises a block room model, comprising a 6-bedded area adjacent to the operating theatre staffed by an additional operating department practitioner. At the beginning of the list, the first and second patients received interscalene brachial plexus blocks with 1% Ropivacaine, delivering rapid onset anaesthesia [22]. Subsequent patients received their anaesthetic block in the time between the cases providing an efficient turnover. None of our patients required conversion to general anaesthesia. The patients were able to observe the procedure intra-operatively and able to participate in a discussion with the team at the same time. Our centre developed this unique approach of inclusive team work and we felt that this led to effective and positive reinforcement for the patient post-operatively. The post-operative rehabilitation was monitored by our extended scope physiotherapists.

Of our three patients who had failure, as defined by recurrent instability symptoms during follow up, two settled with conservative treatment by 12 months. One patient had to undergo open repair for ongoing instability. None of the failures were attributable to primary implant failure. Our post-operative OISS at 12 months indicates an overall excellent outcome. The mean range of abduction was 134.2 ± 6.32 and external rotation was 72.55 ± 5.42 in 60–90 position and is comparable to other studies [23]. All our successful patients were able to return to full time physical activity at 12 months following surgery with no subjective instability.

Prior to the use of suture tape, the standard practice was to use 3 sutures with 3 bone anchors for a standard antero-inferior stabilisation. The cost of tape is comparable to sutures yet the superior shift and hold of the tape led the surgeon to use only 2 tapes and anchors in 50 cases saving 1/3 of the previous implant cost and reducing operative time by the time taken to insert an anchor. This suggests there may also be economic benefits that deserve further study.

## Conclusions

In summary, our results following arthroscopic anterior shoulder stabilization with bone anchors and 1.5 mm suture tape using awake anaesthesia has been reliable and provides excellent clinical outcomes comparable with the available literature for this type of surgery.

The limitations of our study are the short term follow-up so far and lack of a clear control group. Our data collection did not include range of motion of the contralateral normal shoulder for post-operative comparison. Although the OISS is well validated in its use, it does not quantify the level of demand placed on the shoulder. The training of the anaesthetist and theatre team in performing interscalene blocks needs to be considered. Further studies to review the long term results and cost implications comparing awake anaesthetic technique versus standard general anaesthesia for anterior shoulder arthroscopic stabilization need further consideration.

## Abbreviations

IGHL: Inferior Gleno Humeral Ligament
MRA: Magnetic Resonance Arthrogram
OISS: Oxford Instability Shoulder Score
PEEK: Poly Ethyl Ethyl Ketone
